# Application of Carbon–Flax Hybrid Composite in High Performance Electric Personal Watercraft

**DOI:** 10.3390/polym14091765

**Published:** 2022-04-26

**Authors:** Jan Zouhar, Martin Slaný, Josef Sedlák, Zdeněk Joska, Zdeněk Pokorný, Igor Barényi, Jozef Majerík, Zdeněk Fiala

**Affiliations:** 1 Institute of Manufacturing Technology, Faculty of Mechanical Engineering, Brno University of Technology, 61669 Brno, Czech Republic; slany.m@fme.vutbr.cz (M.S.); sedlak@fme.vutbr.cz (J.S.); fiala.z@fme.vutbr.cz (Z.F.); 2 Department of Mechanical Engineering, Faculty of Military Technology, University of Defence in Brno, 66210 Brno, Czech Republic; zdenek.joska@unob.cz (Z.J.); zdenek.pokorny@unob.cz (Z.P.); 3 Department of Engineering Technologies and Materials, Faculty of Special Technology, Alexander Dubček University of Trenčín, 91101 Trenčín, Slovakia; igor.barenyi@tnuni.sk (I.B.); jozef.majerik@tnuni.sk (J.M.)

**Keywords:** flax, hybrid composite, personal watercraft

## Abstract

Within the herein presented research, we studied the applicability of flax fabrics for composite parts in personal watercrafts in order to enhance damping of vibrations from the engine and noise reduction (which is relatively high for contemporary carbon constructions). Since the composite parts are intended to be exposed to humid environments requiring high levels of mechanical properties, a carbon–flax composite was selected. Samples of carbon, fiberglass, flax, and hybrid carbon–flax twill and biax fabrics were subjected to tensile and three-point bending tests. The mechanical properties were also tested after exposure of the samples to a humid environment. Damping was assessed by vibration and noise measurements directly on the complete float for samples as well as real parts. The hybrid carbon–flax material exhibited lower values of tensile strength than the carbon material (760 MPa compared to 463 MPa), but, at the same time, significantly higher than the other tested materials, or flax itself (115 MPa for a twill fabric). A similar trend in the results was observed for the three-point bending tests. Vibration tests and noise measurements showed reductions in vibration amplitude and frequency when using the carbon–flax hybrid material; the frequency response function for the watercraft part assembled from the hybrid material was 50% lower than for that made of carbon. Testing of samples located in a humid environment showed the necessity of surface treatment to prevent moisture absorption (mechanical properties were reduced at minimum by 28%). The tests confirmed that the hybrid material is satisfactory in terms of strength and its contribution to noise and vibration damping.

## 1. Introduction

Natural fibre reinforced composites have become popular, especially in the automotive and construction industry. Given their favourable price and weight, they are very attractive for the production of automobile and aerospace components, bicycle frames, window frames, sports equipment, etc. [1]. Furthermore, environmental efforts encourage the use of extremely lightweight materials to decrease the fuel consumption of vehicles, leading to reduction of carbon dioxide emissions. Therefore, glass fibres (very popular in the past) are becoming less attractive because of their weight and recycling difficulty. On the other hand, flax and other natural fibres open new opportunities for biocomposites with high stiffness/weight ratio and better recyclability [2,3]. Compared to carbon fibre composites, they also offer better damping properties. The greatest weakness of natural fibre composites is, however, their lower mechanical performance, which is limiting for their application [4,5]. Peças et al. [6] presented a comprehensive review on properties of selected natural fibres used as reinforcements within composite materials. They identified flax fibres to be among the most suitable for the automotive industry, sports equipment, or civil engineering. Flax fibre reinforced composites have been used since the late 1930s, but their industrialization increased over the last years. In addition, the manufacturing steps—flax growing and cultivation, agricultural methods, retting methods, fibre extraction techniques—have evolved in time, which enhanced the properties of final products [7]. Kandemir et al. [8] studied physical, thermal, and mechanical properties of four types of natural fibres–namely jute, kenaf, curaua and flax–and reported that curaua and flax exhibited advantageous mechanical properties comparable with those of glass fibre reinforced polymer composites.

Researchers often discuss the advantages of glass and flax fibre reinforced composites, not only from the environmental viewpoint. Poilâne et al. [9] investigated the room-temperature yield point of these two very popular fibres, and documented that flax fibre reinforced polymers exhibited plastic behaviour after a short period of elasticity. Furthermore, flax fibre reinforced composites feature specific characteristics which cannot be characterized by unique values due to their strong dependence on the measurement method (e.g., density) [10]. Therefore, the selected testing method should take into consideration the real conditions under which the product is intended to be used.

Although natural fibre reinforced composites feature (not only) the abovementioned positive aspects, they are mostly produced from twisted yarns of short natural fibres, which cause problems with their impregnation. Poor impregnation increases porosity and deteriorates mechanical properties [11]. Besides water sensitivity, variability in mechanical performance is a disadvantage of natural fibres. Nevertheless, researchers have developed treatments to eliminate the mentioned problems, e.g., Whitacre et al. [12] presented positive effects of zein protein treatments on improving mechanical properties of flax fibre reinforced composites. Another solution is hybridization; hybridization of natural reinforced fibre composites by incorporation of carbon fibres has positive effects on properties of the final composites. Fehri et al. [13] proved that carbon plies applied near the surface decrease porosity; the study showed that a single carbon ply placed on the surface decreased the diffusion coefficient to half its value and the water content by 40%.

Generally, hybridization enhances properties of reinforced composites by using more than one reinforcement under the same matrix [14]. Atmakuri et al. [15] analysed mechanical properties and wettability of hybrid composites, and discovered that hybrid composites showed better mechanical properties–especially higher flexural properties–than pure flax composites. Bolcu et al. [16] confirmed these findings while studying mechanical properties of composites with dammar based hybrid matrices. They also reported advantageous vibration damping of the analysed composites. Another important but often neglected characteristics is water ageing behaviour, which can decrease composite stiffness, cause matrix crosslinking, or layer interfaces release. This behaviour is generally more favourable for hybrid composite than for flax fibre reinforced ones [17]. The ability of hybrid carbon–flax reinforced composites to improve damping properties of vehicles opens new possibilities in automotive, marine, and vehicle engineering in general. Fairlie and Njuguna [18] discovered that adding an external flax layer can increase the damping ratio of a composite by more than 50%, while adding two layers imparts increase by more than 90%. However, Mahmoudi et al. [19] documented that damping properties are related to fibres’ orientations. Among natural fibres, pre-impregnated flax fibre textiles (also called prepregs) featuring enhanced mechanical properties can also be used [20].

There are numerous applicable production techniques for flax fibre reinforced composites. Compression moulding, often combined with hot pressing, is a widely used technology for manufacturing natural fibre reinforced composites. Symington et al. [21] presented a vacuum infusion rig—another technology ensuring consistent quality of composites. Vacuum Assisted Resin Transfer Moulding (VATRM) and Seemann Composite Resin Infusion Moulding Process (SCRIMP) are other technologies with positive impacts on mechanical properties of natural fibre reinforced composites. Especially VATRM has become very popular in recent years due to its high productivity and low operational costs. However, VATRM introduces defects, such as voids, which deteriorates quality and mechanical properties of produced composite parts [22]; the content of voids is especially important since it can negatively influence mechanical properties of fibre reinforced composites. Therefore, from a quality viewpoint, monitoring this parameter is crucial, especially due to the development of modern manufacturing techniques (e.g., VATRM, out-of-autoclave (OaA), automated prepreg laying, etc.) introducing lower production costs and shorter manufacturing times [23,24]. For example, Kedari et al. [25] suggested to increase mould temperature and vacuum and appropriately reduce inlet pressure to produce high quality VARTM parts.

Testing and evaluation of properties of both fibres and matrix according to a wide range of criteria is important for the manufacturers to select the most suitable combinations of reinforcements and matrices for their final composite products. AL-Oqla et al. [26] emphasized the importance of combined economic, environmental, and technical viewpoints to achieve better composite performance. Researchers have massively studied tensile and compressive mechanical properties of flax fibre reinforced composites. Besides standard mechanical tests, damaged response through SEM (Scanning Electron Microscope) is often observed in order to describe in-plane modulus and inelasticity evolution [27,28,29,30,31]. SEM is suitable to perform morphological analyses of composites reinforced with wires and fibres [32,33], and can advantageously be combined with other methods such as Y-ray microcomputed tomography to analyse influence of water absorption on the behaviour of flax and glass reinforced hybrid composites [34]. With regards to mechanical testing, the single fibre tensile test, dry fibre bundle test, or impregnated fibre bundle test (IFBT) are common methods [35,36,37]. The IFBT method is also suitable to test the effects of individual extraction and refining steps on stiffness and strength of natural fibres in composites [38]. For deeper understanding of mechanical behaviour and damage modes, acoustic emission evaluation–possibly combined with other modern methods such as post-mortem microscopy or neutron diffraction–can be used [30,39,40,41]. The output is a frequency response function (FRF) based on vibration response data signals acquired by one or more microphones [42]. The FRF offers a set of linear equations that are solved by a bounded-variables least-squares algorithm. The minimum number of natural frequencies and mode shapes used to compute FRF matrices is three [43,44].

The real component that the presented material is planned to be used for is a part of Jetsurf Electric, an electric-powered motor float by MSR Engines. Currently, the float structure is made of carbon fabric and consists of approximately 30 parts that are glued or mechanically joined together. The requirement is to reduce vibrations and noise affecting the rider and the surrounding environment while maintaining mechanical properties and favourable weight. Available studies primarily focused on the effects of drive system on vibrations [45,46,47]; however, they did not consider the mechanical bond to the watercraft hull. Due to the internal construction of the float, the battery, and the drive system in which they are located, sandwich structures cannot be used since they are typically very thin and thus prone to quick failure due to the dynamic load and activity of the rider and surrounding waves [48]. Therefore, thin-walled parts with a thickness of up to 2–3 mm have to be used. In case of flax materials and fabrics, the majority of available studies focused on materials with similar thickness, but only evaluated selected material properties [49,50,51]. For example, only a few researchers studied the behaviour of variable twill-like fabrics via mechanical properties and damping characteristics [49,50,51,52,53]. For complex characterization of properties, various kinds of fabrics (twill, biax, UD, etc.) by a single producer should be evaluated from the viewpoint of mechanical properties, and subsequently tested for application in hybrid fabrics. Furthermore, the composite fabric should be symmetrical, since it is planned to be produced using autoclaves—uniform composition is necessary to eliminate temperature stress and subsequent deformation, i.e., springback, after curing [54]. Moreover, the effect of a humid environment should be tested [54,55]. The component selected for the herein-presented study was a cover of the engine compartment of an electro-motor.

## 2. Materials and Methods

### 2.1. Materials

For the purposes of testing and production of tested parts, a range of materials commonly used in construction applications was chosen. The chosen fabric types were Twill and Biaxial with a basis weight of about 200 g/m^2^. The area density was chosen based on their spread in practical applications, favourable handling, and availability. The properties of the used fabrics can be found in Table 1. Flax fabrics were by B-Comp Ltd. (Fribourg, Switzerland); the others were by GRM Systems ltd (Olomouc, Czech Republic).

**Figure 1:**
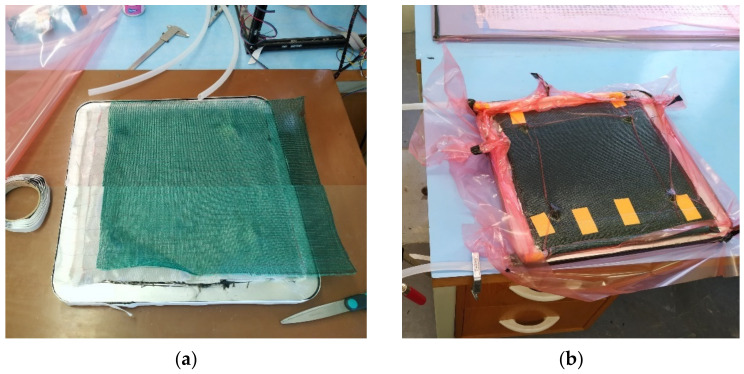
Vacuum infusion process: (**a**) prepared auxiliary materials; (**b**) product during curing.

Boards for testing purposes were made from these materials via the Vacuum Infusion (VARTM) method [56,57], which is widely used for production of high-quality composites with all types of weaving, and guarantees constant conditions of saturation, curing, control, and processing. Examples of the board production process are shown in Figure 1a,b. LG700 epoxy resin with HG700 hardener (as used by the manufacturer—GRM Systems, Olomouc, Czech Republic) were used for the matrix; this is suitable for Resin Transfer Moulding (RTM) and infusion applications. The resin is used in a weight ratio of 100:30. The mechanical properties of the unreinforced resin are tensile strength of 65–75 MPa, elongation at break of 6–8%, and flexural strength of 110–120 MPa [58]. All the samples were cured at 24 °C for 24 h before demoulding. The samples were cut after five days, and no additional post-cure process was required.

Boards with the dimensions of 300 mm × 300 mm, which were subsequently cut to the dimensions of 220 mm × 220 mm to measure vibrations, were produced for attenuation testing. Samples for the tensile tests and three-point bending tests were produced according to the given EN ISO 527-4 and ISO 14125: 1999 standards. To measure vibrations, boards of two thicknesses with four and eight layers of material were made; the layers were laid to form a balanced symmetrical laminate (0/90; ±45) S with quasi-isothropic properties [59]. Thicknesses and other properties are summarized in Table 2. The weight fraction according to [59,60] and other properties were calculated for the resulting laminates.

**Figure 2:**
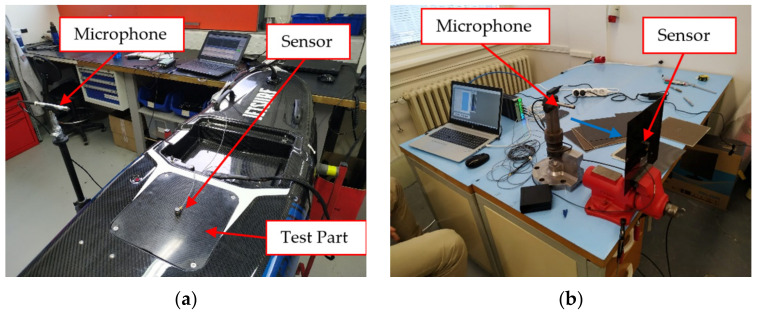
Frequency measurement: (**a**) laboratory measurement setup; (**b**) real measurement of float setup.

The part for testing in the real environment—engine cover—was also made using the vacuum infusion technology (Figure 1), and can be seen in Figure 2a (the cover of the float engine compartment is located in the sensor area). The composition of the part was determined in accordance with the sample tests; three types of samples (D01-D03) were produced. Sample D01 was made of four layers of 200 g/m^2^ fabric with a 2 × 200 g/m^2^ inner flax insert ending 20 mm from the edge to prevent water penetration into the flax area. The edges were complemented with carbon fabric to maintain constant thickness, and the weight was 273 g. Sample D02 was made to be identical to sample D01, but with complete flax liner (reaching to the edges); the sample weight was 223 g. The composition of sample D03 consisted of four layers of GG600T fabric (Deltapreg, Sant’Egidio alla Vibrata, Italy) weighing 600 g/m^2^; sample weight was 265 g. After production, the samples were cut to required shapes using a KUKA KR 60HA robot with a machining spindle and a sintered carbide tool with diamond coating.

### 2.2. Testing Equipment

Tensile strength measurement was performed using a ZwickRoel Z100 measuring machine according to EN ISO 527-4 standard (the main parameters and size of the sample corresponded to the standard) [61]. The samples were 25 mm wide, 210 mm long, and 1.05 mm thick, and were fixed in the jaws with glass fibre attachments using an epoxy glue. Five valid samples were measured in each measurement series. The loading speed was set to 2 mm/min. Flexural tests were measured according to EN-ISO 14125: 1999 [62] standard with the help of the same ZwickRoel Z100 device using reversal and jig. The length of the samples corresponded to the standard according to the thickness and distance of the supports; the width of the samples was 15 mm Five samples were again measured for each material. The measuring jig allowed to change the distance of the supports as described in the standard, so that samples with different thicknesses (varying according to the production method) could be measured. The loading speed was set to 2 mm/min.

Conditioned samples were prepared for testing in a humid environment. The conditioned samples were placed in a Constazo KB300 climate chamber heated to 40 °C with 100% humidity for 100 h. The conditions simulated the above-described real load. The samples were then removed and within a few hours subjected to tensile and deflection testing. Among the results of the measurement were the maximum values of strength, modulus, and strain.

As regards morphology and damage assessment, SEM Tescan Mira 4 equipment was used to monitor the fractured surface to characterize failures of the individual materials. In order to study and compare the behaviours of the composite materials, ARAMIS digital image correlation system enabling to accurately evaluate the behaviour of a material under load in time was used [63,64,65].

Evaluations of RMS (Root Mean Square) mechanical vibration, noise levels, and dynamic properties were also made to find out the most suitable composite material for the Electric Personal Watercraft. Measurement of FRF (frequency response function) was chosen to evaluate dynamic properties of the samples. Modal analyses were not performed because investigating the samples’ mode shapes was more suitable to compare the results of real measurements with mathematical models. Brüel & Kjaer Photon + equipment was used to perform the experimental measurements (see Table 3). The FRFs measurements were performed in laboratory conditions. A bench vice was used to clamp the composite samples (see Figure 2b). Keeping identical positions of samples in the vice, the position of accelerometer and hits by modal hammer were emphasized when exchanging the samples. The 4517-type accelerometer was chosen since its low weight does not affect the FRFs results and low sensitivity prevents sensor overloading. Each FRF peak represented a frequency the system in which vibrated excessively. This function could be used to calculate Young’s modulus, loss factor, and damping ratio at different resonant frequencies of each specimen [18].

## 3. Results and Discussion

### 3.1. Tensile Properties

**Figure 3:**
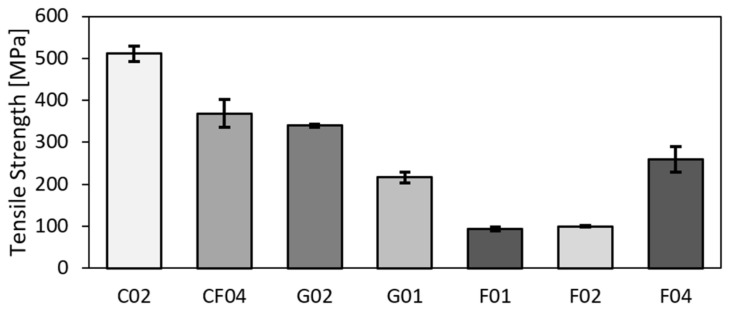
Tensile strengths of test samples.

**Figure 4:**
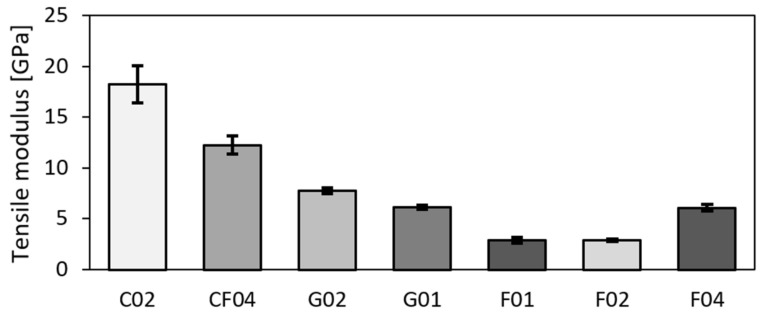
Tensile moduli of test samples.

The results of tensile tests are summarized in Table 4. The parameters of tensile modulus, tensile strength, and fail strain were further calculated from the tensile test records. The final values are the arithmetic mean values acquired from five samples with the standard deviation *N* = 5. The C02-carbon composite material exhibited the highest values of both the tensile modulus and strength. The hybrid material also had very favourable properties and exhibited higher values than the glassy and pure flax materials. Tensile strength for all the samples is depicted in Figure 3, whereas the comparison of tensile moduli is shown in Figure 4. From Figure 3, it is evident that the flax composite featured significantly lower properties than glass for both the twill and biaxial materials. In general, the twill–woven material exhibited lower values of both the tensile modulus and strength than the biaxial material. Compared to the unidirectional material flax with fibres in the direction of the applied force, the twill glass fabric achieved less advantageous properties. As regards the tensile modulus (Figure 4), the fabric and bi-axial types of both the flax and glass composite exhibited minimum differences.

**Figure 5:**
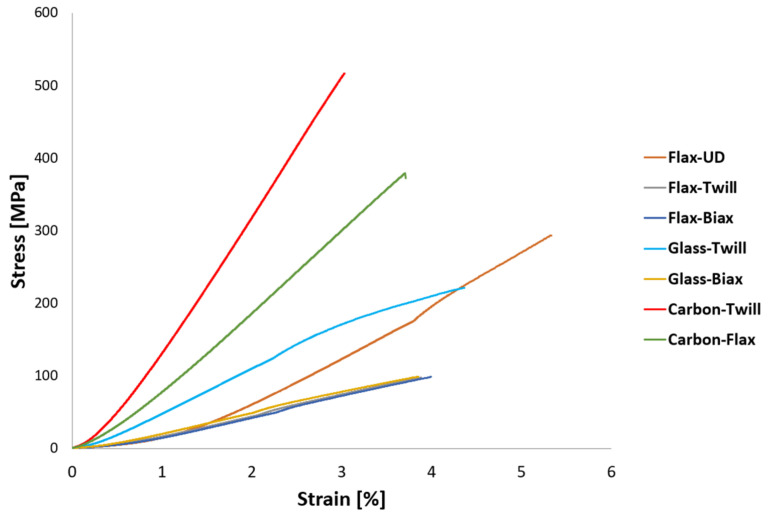
Tensile test results, stress-strain curves for test samples.

However, differences are more evident from Figure 5, showing graphical depictions of the tensile tests results, i.e., stress-strain curves, the tensile moduli from which were derived. As for the glass materials, the tensile strength was higher for the biaxial fabric, but the shapes and slopes of the curves revealed that their tensile moduli were comparable. This phenomenon is well-described in the literature and is typical for composite materials, the value of tensile modulus for which is not entirely meaningful without knowing the shape of the stress-strain curve. For example, Zhang [65] documented such mechanical behaviour for glass–flax hybrid materials. In regards to the other materials, the results for the carbon composites and hybrids were evidently the most advantageous, while the lowest values were achieved for the flax (note that the courses of the load curves for the fabric and biaxial materials were almost identical). The values of strain were primarily given by the composition of the samples, which was quasi-isotropic. Therefore, the strain was of higher values for the one-way samples, or samples arranged in ideal directions. All the evaluated samples experienced final fracture, as can be seen from the curves in Figure 5.

The values of tensile strength and modulus presented in available literature can significantly differ according to the type of the used fabric, epoxy resin, and fabrication method [20]. The tensile test results herein acquired for the hybrid composite can be compared to those reported by Fairlie et al. [18] and Al-Hajaj [49], who evaluated hybrid composite, pure carbon, and flax. The compositions of the tested samples were comparable, and the differences in the tensile strength were within the standard deviation. The tensile modulus was higher for the base samples, which can be attributed to different fabric composition in each layer. AL-Hajaj [49] studied a flax fabric in an UD form with 0/90 or ±45° layering, and presented differing results for different layering directions of the flax fabric within the composites. Comparing to the herein presented quasi-symmetric flax fabric, the values of both the tensile strength and modulus were higher for the 0/90 layering direction, and comparable or lower for the ±45° layering direction. This can be attributed to usage of UD fabrics instead of twill-like ones. The results acquired for the flax fabric can be compared to studies [15,16,27].

### 3.2. Flexural Properties

**Figure 6:**
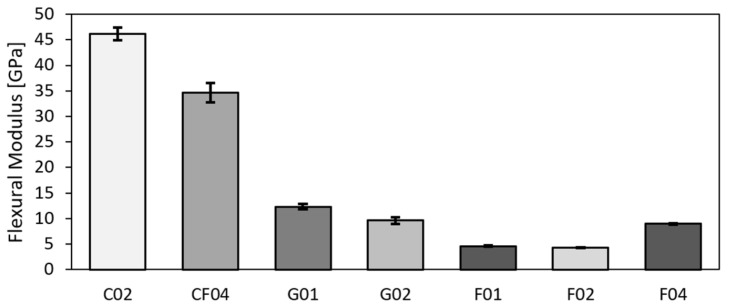
Flexural moduli of test samples.

**Figure 7:**
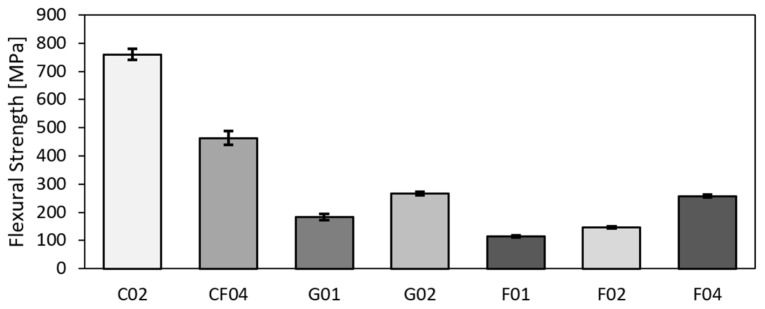
Flexural strengths of test samples.

The values acquired based on the three-point bending test, i.e., flexural modulus, flexural strength, and flexural strain, are given in Table 5. The values are again the arithmetic means from five samples with the standard deviation *N* = 5. Similar to the tensile test results, the carbon sample, together with the hybrid one, exhibited the highest values. However, the hybrid and glass samples exhibited differences. The values for the hybrid sample corresponded to those acquired for the carbon ones, while the values for the glass reached similar values as for the flax (see Figure 6 depicting the tensile strengths and Figure 7 showing the tensile moduli). The results from three-point bending testing reveals that the glass fabric exhibited very low flexural strength—compare 183 MPa to 760 MPa acquired for the carbon and 463 MPa reached for the hybrid fabric, respectively. The trend was similar also for the flexural modulus—compare 12.30 MPa for glass, 46.12 MPa for carbon, and 34.63 MPa for hybrid carbon–flax materials, respectively. Similar values for comparable materials were reported by others [20,65,66]. The comparison of the results also shows that the flexural strength and moduli acquired for the flax UD were comparable to those acquired for the glass bi-axial fabric.

**Figure 8:**
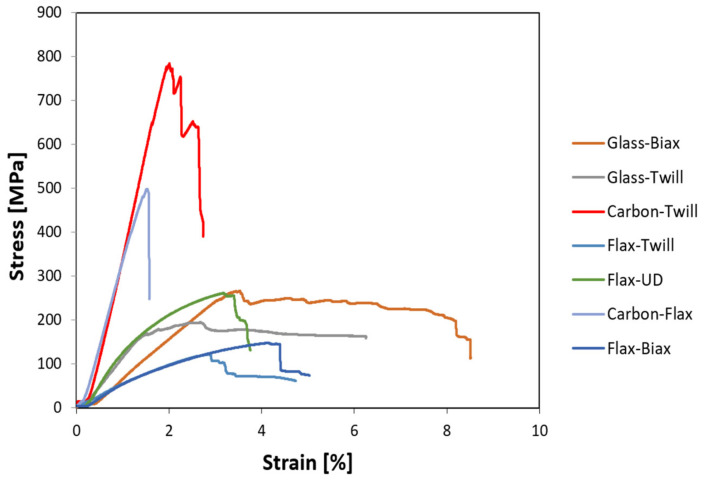
Flexural test results stress-strain curves for test samples.

From the flexural stress-strain curves depicted in Figure 8, it is evident that after reaching the maximum flexural stress (force), the samples further deformed at lower loads, but with no final fractures. This phenomenon was the most evident for the glass and flax samples [50]. For each sample, the test was stopped after 30% loss of the maximum reached force. The flexural moduli values were derived from the curves—the phenomenon mentioned in Section 3.1 was also evident here; when the flexural stress for the glass–biax sample reached higher value than for the glass-twill sample, its flexural modulus value was lower, given by the slope of the curve acquired for the highest achieved stress. Interesting behaviour was observed for the glass-based fabrics—the values of strain corresponding to the maximum stress values were relatively high, up to approx. 8% (also observed e.g., in [67]). However, such high imposed strain already introduces irreversible changes, and the maximum observed plasticity of the composites is thus not practically useful (typically usable up to 2.5–3% of strain). In regards to the comparison of twill and biax types of fabrics with identical bases, the courses of the stress-strain curves were similar (Figure 8).

Carbon–flax composites are sometimes prone to matrix degradation, which is common for composites with resin surplus. This phenomenon is related to the brittleness of epoxy resins, which can only undergo limited deformation before failure, and thus feature low impact strength [66]. Furthermore, cracks spread quickly from the flax core of such hybrid material towards the carbon layers (featuring lower resin fraction than the flax core) [65].

### 3.3. Conditioned Samples

Conditioned samples were tested only for flax UD, flax-biax, and hybrid materials. Other materials–glass and carbon–are standard, and their environmental degradation values are known [59,60]. When impacted by moisture, the thickness of the tested samples increased as follows: by 11.8% for the flax UD sample, by 13.9% for the flax biax sample, and by 2.34% for the carbon–flax one. The evident difference observed for the carbon–flax sample could be attributed to the effect of the epoxy inter-layer located between the flax core and carbon cover. The inter-layer gets damaged, i.e., micro-delaminated, by surrounding humidity and different wettability of the individual layers (also documented e.g., in [56,67]). Furthermore, fibres soaked with moisture compress the surrounding epoxy resin and contribute to the formation of micro-cracks.

**Figure 9:**
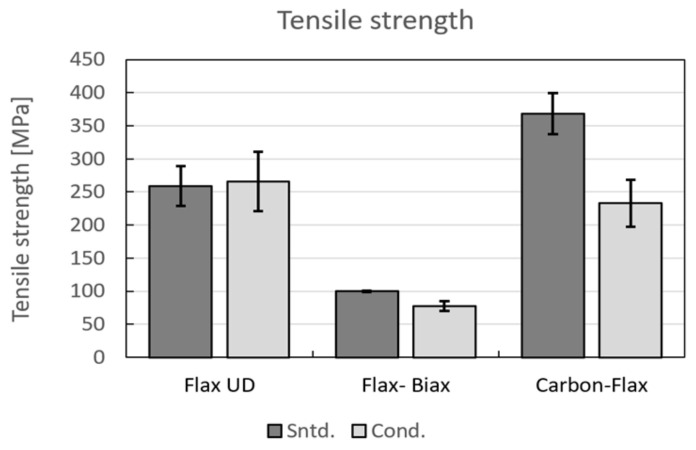
Tensile strengths of conditioned samples.

From the tensile tests, the results of which are summarized in Figure 9, it is evident that the standard deviation increased for all the flax samples. Moreover, the change in tensile properties was minimal except for the hybrid composites, for which values 28% lower were achieved (compared to standard samples). This can mainly be attributed to the effect of moisture on materials’ interface, i.e., location in which failure was initiated. These conclusions are supported by study [68] reporting a correlation between increase in material thickness, decrease in mechanical properties, and occurrence of failure.

**Figure 10:**
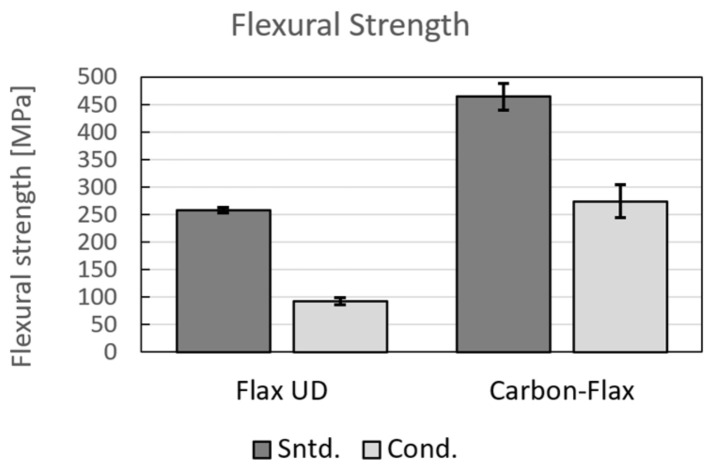
Flexural strengths of conditioned samples.

The UD flax material and hybrid fabric were tested by the three-point bending test (see Figure 10 for the flexural strength results). Similar to the tensile test results depicted in Figure 9, the results of bending tests showed that moisture decreased properties of the hybrid material. However, the decrease in the values achieved for the Flax UD sample was even more noticeable (more than 60%). Comparable results were acquired by César dos Santos et al. [54], who achieved the flexural strength of 248.02 ± 26.24 MPa for a longitudinal specimen, and the flexural strength of 77.93 ± 8.25 MPa for a specimen placed for four days in a 100% humidity environment (which is in agreement with the herein performed experiment). In the presented study, the hybrid fabric still exhibited higher values given by the carbon layer. The core itself behaved as a sandwich structure primarily loaded by shear stress. Therefore, the drop in the flexural values was not as large as for the flax UD sample [34].

### 3.4. Frequency and Noise Characteristics

**Figure 11:**
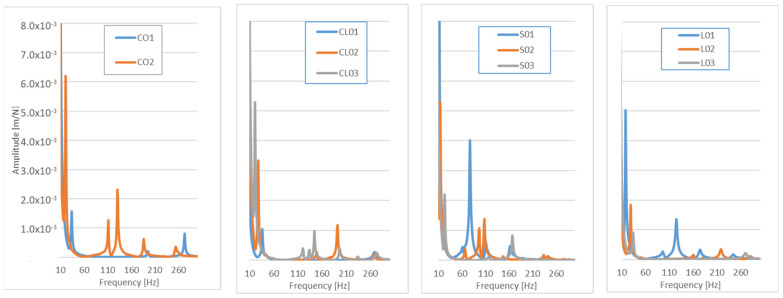
FRF measurement of composite samples: CO—Carbon, CL—hybrid carbon–flax, S—glass, L—Flax.

A bench vice is not the most favourable solution to fix samples, however, all the samples were tested under identical conditions. The first FRF measurement showed that the carbon samples featured the highest amplitude values at higher frequencies when compared to the other tested materials exhibiting lower amplitude values at lower frequencies (Figure 11). The thickness and composite density are the most relevant parameters having significant impact on dynamic properties of the sample. Compared to the carbon and glass samples, both the hybrid and flax samples exhibited better attenuation values; the flax sample exhibited the most favourable properties. The results of frequency analyses were in agreement with previously published research by Chinnasamy [69] and [42,43], who evaluated sisal and jute fibres via the FRF function. A hybrid flax–carbon composite was tested by Fairlie [18]. He confirmed the relation of reduction in damping and the amount of flax content within the composite. However, he used different measurement methods and measured samples. Results for different carbon–carbon combinations can also be found in the work by Assar et al. [52]; they performed the comparison of bending modulus vs. specific damping for a material consisting of components identical to those used in the presented work.

Dynamic behaviour can be determined via FRF measurement, or by modal analysis. At present, there is no available study reporting the behaviour of functional parts made of various composites from the viewpoint of dynamic properties. Singh et al. [53] used FRF measurement to study the effect of epoxy resin content on frequency and damping characteristics of composite samples. Hassani [70] focused on detection of structure defects within composite materials via FRF measurement. Nevertheless, the published studies mostly deal with theoretical studies and focus on the development of mathematical models to predict and characterize dynamic behaviour of various composite materials (e.g., [71,72,73]). Due to this, the herein acquired results cannot be directly compared with results reported by other researchers dealing with composite materials.

**Figure 12:**
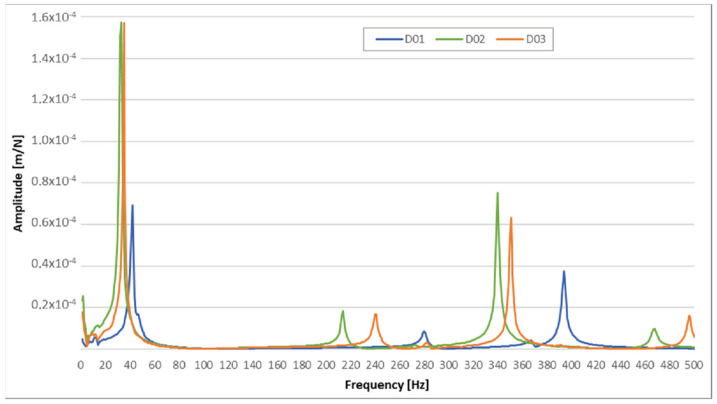
FRF values for final prototypes.

Based on the acquired results, the decision to assemble the final part of the prototype from the flax hybrid composite was made. The final prototype of the selected part was tested whilst directly mounted to the electric personal watercraft (see Figure 2a), as well as clamped to the bench vice. The RMS and sound noise levels for the samples were measured when mounted on the watercraft, and FRFs were measured when camped to the bench vice. The FRFs measurement showed that the best results were acquired for the D01 sample; the differences between the amplitudes and dominant frequencies for the D02 and D03 samples were negligible (Figure 12).

**Figure 13:**
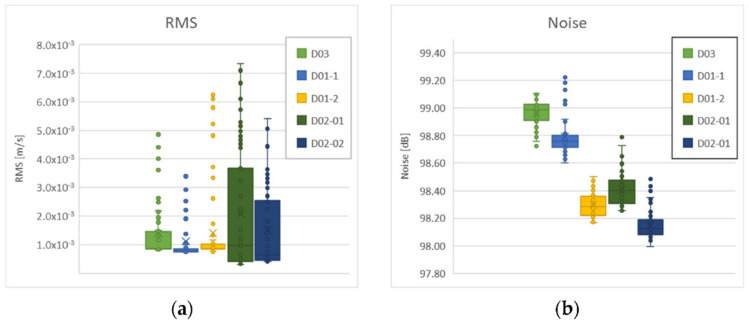
RMS measurement results (**a**); noise level measurement results (**b**).

Final testing of the prototype parts mounted on the watercraft were performed for the maximum revolutions of the engine, i.e., 7200 RPM. The RMS and noise level were calculated for the frequency range of 0–3.2 kHz. The accelerometer was fixed by a special wax in the middle of the tested sample, see Figure 2a. The results of noise level are not conclusive because the prototype part is a small subcomponent that did not affect the results in a greater extent. The problems were instability of engine-speed fluctuations and poor measurement repeatability. The results of RMS measurement are shown in Figure 13a, and the noise levels are depicted in Figure 13b. The most advantageous RMS results were acquired for the D01-1 and D01-2 samples. This fact was in accordance with the results of the FRFs measurements of samples clamped in the bench vice. Assessment of noise level is typically performed under different conditions during on-water operation (especially for diesel engines) [74]. The herein acquired noise level was higher than recommended [75], which can be attributed to the fact that the measurement was performed at high RMPs (more than 80% of maximum) in a closed room. The distance of the microphone and recorded frequencies could also have affected the result.

### 3.5. Microstructure

**Figure 14:**
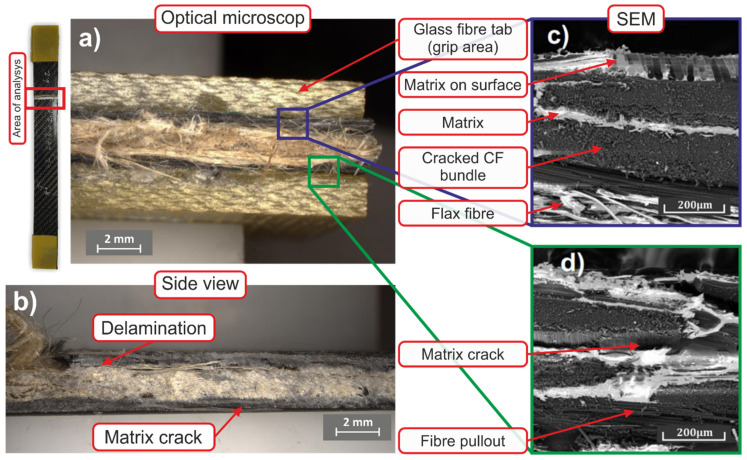
Microstructure of cracked tensile test sample—optical microscopy and SEM analysis, (**a**) top view, (**b**) side view (**c**) SEM image fracture area carbon–flax, (**d**) SEM image carbon bundle–matrix.

**Figure 15:**
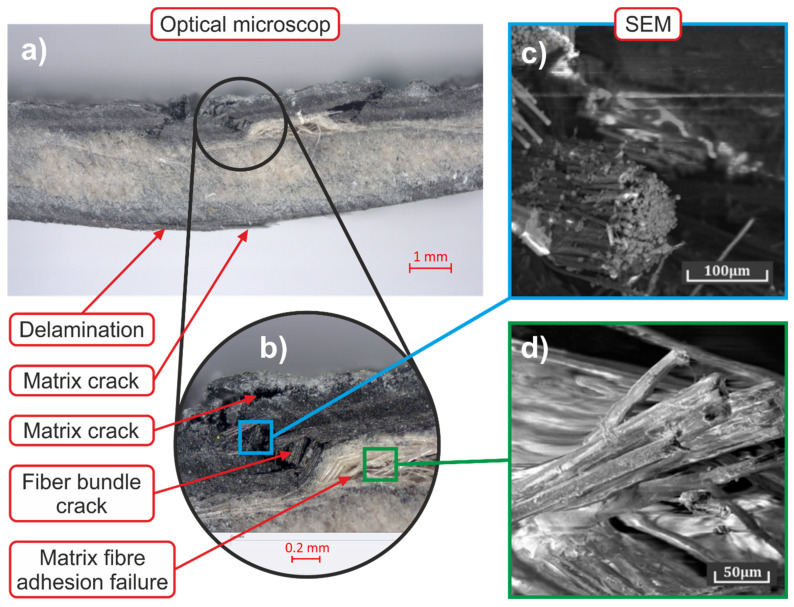
Microstructure of cracked flexural test sample—optical microscopy and SEM analysis, (**a**) side view, (**b**) detail of compressive stress area, (**c**) SEM image of carbon bundle, (**d**) SEM image flax fibre.

Microstructure of the fibre–hybrid sample and fractured surface after tensile test are depicted in Figure 14. The up-view on the fractured surface of the sample depicted in Figure 14a also shows saddle clamps on both ends of the sample. Figure 14a,d depict carbon fibres separated from the matrix, which indicates inter-laminar failure and separation of the matrix from the fibres at crack initiation [27]. Figure 14b shows the side surface of the sample with evident delamination in the carbon—flax inter-layer. A crack in the resin propagating along the borders of the fibres is evident at the bottom side of the sample. Figure 15c shows the interfaces of carbon and flax areas. As can be seen, the carbon fibres were not separated from the matrix and there was no tearing of the material. It can be assumed that crack development primarily occurred at inter-laminar carbon regions—at resin–fibre interfaces—and propagated towards the flax region, in which the individual fibres were torn due to their larger size and poor fibre-matrix interfacial properties [31]. The results correspond to those acquired by Dinesh el al. [51], who documented poor interfacial adhesion and delamination for a flax–carbon hybrid composite in tension, compression, and during bending. Increasing the content of flax fibres promotes the occurrence of fraction without delamination.

The fractured surface after bending is depicted in Figure 15. Figure 15a depicts a region featuring the delamination and matrix crack of carbon fibres, pointing to the occurrence of tensile stress at the bottom of the sample. On the other hand, compressive stress occurs at the top of the sample (Figure 15b)—its morphology exhibiting brittle fracture of a carbon fibre bundle is shown in detail in Figure 15c. Given their deteriorated cohesion with the matrix [27], the flax fibres were primarily damaged by delamination and massive failure of the matrix with the fibres (evident in Figure 15d). Such separated fibres could be observed in the whole fractured area, as depicted in Figure 15b.

## 4. Conclusions

The study focused on the assessment of the suitability of usage of carbon–flax hybrid materials for prospective production of composite parts for personal watercrafts via fundamental mechanical testing. The hybrid material exhibited advantageous damping of vibration and noise, while maintaining favourable mechanical properties, weight, and transversal dimensions of the part. The carbon–flax composite achieved 72% of tensile strength and 60% of flexural strength when compared to the values acquired for a carbon sample (CF04). Other materials, such as pure flax and glass, achieved much lower values (below 35%). Decrease in the flexural strength of carbon–flax samples exposed to a humid environment (simulating realistic service conditions) was relatively low—only by 40% (for the pure flax UD sample it was up to 64%). This phenomenon requires further study to optimize the surface treatment in order to avoid moisture absorption while maintaining favourable mechanical characteristics. Based on the performed tests, real parts were fabricated and tested in operation. The carbon–flax composites achieved lower RMS and damping for both the samples and real parts during operation. The reduction of noise can be noticed by the electric board rider.

## Figures and Tables

**Table 1:** Fabrics used for samples.

Fabric	Style	Weight [g/m^2^]	Thickness [mm]
E-Glass	Bi-Ax	200	0.33
E-Glass	Twill 2/2	200	0.29
Flax	Bi-Ax	350	0.62
Flax	Twill 2/2	200	0.45
Flax	UD	280	0.35
Flax–Carbon	UD	225	0.32
Carbon	Twill 2/2	200	0.25

**Table 2:** Laminates and their properties.

Laminate ^1^	Style	Stacking Sequence	Thickness [mm]	Composite Density [g/cm^3^]	Fibre Volume Fraction [%]
C01	Twill 2/2	8×C	2.02 ± 0.03	1.47	42.12
C02	Twill 2/2	4×C	1.05 ± 0.02	1.39	42.74
CF01	UN	8×C+F (hybrid)	2.65 ± 0.02	1.30	31.74
CF02	Twill 2/2	C+C+F+C+C	1.48 ± 0.05	1.29	49.19
CF03	Twill 2/2	C+C+1/2F+C+C	1.03 ± 0.04	1.50	55.91
CF04	Twill 2/2	C+C+F+F+C+C	2.25 ± 0.04	1.42	51.20
G01	Twill	4×G	1.10 ± 0.03	1.55	38.51
G02	Bi-axial	4×G	1.31 ± 0.02	1.76	46.13
G03	Twill 2/2	8×G	2.02 ± 0.03	1.70	38.86
F01	Twill	4×F	1.8 ± 0.05	1.11	31.12
F02	Bi-ax	4×F	2.65 ± 0.06	1.19	39.64
F03	Twill	8×F	3.50 ± 0.07	1.21	29.11
F04	UD	8×F	2.60 ± 0.04	1.22	51.10

^1^ C—Carbon; CF—Carbon–flax hybrid; G—Glass; F—Flax.

**Table 3:** Brüel & Kjaer measuring equipment.

Analyzer	Accelerometer	Microphone	Modal Hammer
Photon+	4517 mini ACC	4189	8204
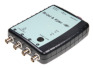	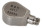	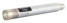	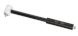
Analog channels: 4 input/2 output Frequency range: 84 kHz Dynamic range: 115 dB	Sensitivity: 1.02 mV/g Frequency range: 20 kHz	Frequency range: 20 kHz Sensitivity: 50 mV/Pa	Sensitivity: 22.7 mV/N Full-scale force range: 220 N

**Table 4:** Tensile properties of samples.

Material	Style	Tensile Modulus [GPa]	Tensile Strength [MPa]	Fail Strain [%]
C02	Twill 2/2	18.21 ± 1.83	511.26 ± 18.31	2.8 ± 0.22
CF04	Twill 2/2	12.24 ± 0.88	368.44 ± 32.90	2.96 ± 0.34
G02	Bi-axial	7.77 ± 0.29	339.24 ± 13.33	4.6 ± 0.18
G01	Twill	6.14 ± 0.21	216.22 ± 3.51	4.03 ± 0.28
F01	Twill	2.86 ± 0.27	93.87 ± 4.44	3.34 ± 0.31
F02	Bi-axial	2.89 ± 0.15	99.9 ± 1.15	3.57 ± 0.09
F04	UD	6.07 ± 0.31	259.12 ± 30.04	4.21 ± 0.24

**Table 5:** Flexural properties of samples.

Material	Style	Flexural Modulus [GPa]	Flexural Strength [MPa]	Flexural Strain [%]
C02	Twill 2/2	46.12 ± 1.24	760.38 ± 18.64	2.01 ± 0.10
CF04	Twill 2/2	34.63 ± 1.91	463.94 ± 23.86	1.58 ± 0.05
G01	Twill	12.30 ± 0.55	183.24 ± 11.01	2.49 ± 0.14
G02	Bi-axial	9.61 ± 0.70	266.41 ± 6.71	3.10 ± 0.67
F01	Twill	4.59 ± 0.13	115.01 ± 4.64	3.08 ± 0.26
F02	Bi-axial	4.28 ± 0.11	146.43 ± 3.21	3.94 ± 0.39
F04	UD	8.96 ± 0.11	257.50 ± 4.91	3.15 ± 0.06

## Data Availability

Not applicable.
